# Protein–Ligand Binding Volume Determined from a Single 2D NMR Spectrum with Increasing Pressure

**DOI:** 10.1021/acs.jpcb.1c02917

**Published:** 2021-05-25

**Authors:** Gediminas Skvarnavičius, Zigmantas Toleikis, Vilma Michailovienė, Christian Roumestand, Daumantas Matulis, Vytautas Petrauskas

**Affiliations:** † Department of Biothermodynamics and Drug Design, Institute of Biotechnology, Life Sciences Center, Vilnius University, Saulėtekio 7, 10257 Vilnius, Lithuania; ‡ Latvian Institute of Organic Synthesis, Aizkraukles 21, 1006 Riga, Latvia; § Centre de Biochimie Structurale, INSERM U1054, CNRS UMR 5048, Université s de Montpellier, 34000 Montpellier, France

## Abstract

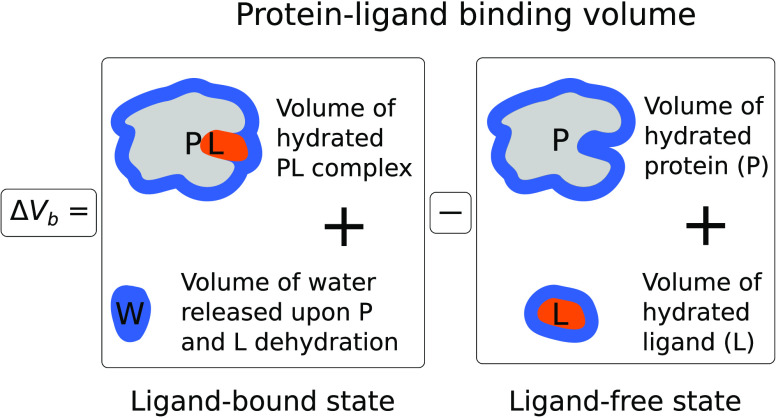

Proteins undergo changes in their partial volumes in numerous biological processes such as enzymatic catalysis, unfolding–refolding, and ligand binding. The change in the protein volume upon ligand binding—a parameter termed the protein–ligand binding volume—can be extensively studied by high-pressure NMR spectroscopy. In this study, we developed a method to determine the protein–ligand binding volume from a single two-dimensional (2D) ^1^H–^15^N heteronuclear single quantum coherence (HSQC) spectrum at different pressures, if the exchange between ligand-free and ligand-bound states of a protein is slow in the NMR time-scale. This approach required a significantly lower amount of protein and NMR time to determine the protein–ligand binding volume of two carbonic anhydrase isozymes upon binding their ligands. The proposed method can be used in other protein–ligand systems and expand the knowledge about protein volume changes upon small-molecule binding.

## Introduction

Most protein–ligand binding studies are performed at ambient pressure and provide the standard Gibbs energy of binding. More in-depth studies often attempt to determine the entropy and enthalpy contributions to the Gibbs energy of ligand binding to a protein. High pressure provides an additional approach to study the protein–ligand binding by revealing the volume changes occurring upon protein interaction with a ligand. This approach provides insight into the changes in the protein partial volume while performing native functions, unfolding, and binding small molecules. These reactions cause the rearrangement of volume-related protein cavities, clefts, and the hydration shell.^1−5^

**Figure 1:**
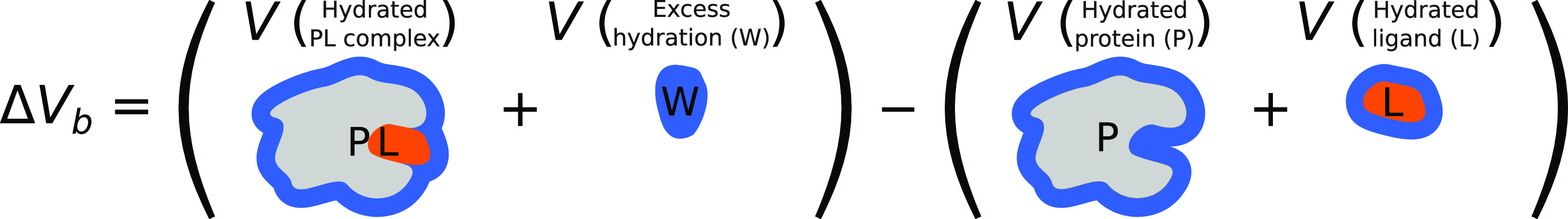
Illustration of the protein–ligand binding volume Δ*V*_b_.^29,32^ W denotes water molecules that the protein (P) and ligand (L) release into bulk water after their partial dehydration upon binding.

High pressure is a key that unlocks various volume-related properties of proteins and reveals their functions. Changes in the protein volume upon unfolding have been extensively investigated.^4,6−13^ High pressure also allowed one to probe structural properties of protein subdomains,^12,14−16^ observe folding pathways and switches between protein conformations,^17−20^ and investigate volume changes that arise upon binding small molecules.^21−31^ As a quantity of the change in the protein volume upon ligand binding, we use a parameter Δ*V*_b_, which is called the protein–ligand binding volume and is defined as the difference between the molar volumes of the bound (hydrated protein–ligand complex plus excess water molecules) and unbound (hydrated protein and hydrated ligand) states. Figure 1 illustrates a simplified definition of the Δ*V*_b_. In the literature, this parameter may have alternative names, such as the reaction or interaction volume.

Various experimental approaches may be used to measure the protein–ligand binding volume including the density and ultrasound velocity techniques,^23,33^ small-angle X-ray and elastic incoherent neutron scattering,^34^ fluorescence spectroscopy at elevated pressures,^24,30,31^ and high-pressure NMR.^16,29,35,36^ The NMR spectroscopy is particularly informative in volumetric measurements because it can monitor changes in the local amino acid rearrangement. This allows identification of the binding-affected amino acid residues and analyze changes at the ligand binding site. Such features are unavailable in density- or fluorescence-based techniques, which provide ensemble-averaged properties, and many details remain hidden. Advantages of NMR spectroscopy come at a price: this assay requires relatively high concentrations of ^15^N-labeled proteins.^37^ The protein–ligand dissociation constant, *K*_d_, can be accurately determined only if the protein concentration is in the range of *K*_d_, and thus the micromolar concentration of a protein in the NMR experiment limits the range of possible ligand affinities to weak and moderate.^38,39^ All mentioned techniques reveal different aspects of protein–ligand interactions and have not only advantages but limitations also. We think that the best way to obtain a complementary view of the protein–ligand binding volume is to use several techniques by exploiting their strengths and overcoming possible weaknesses.

In this study, we used recombinant human carbonic anhydrase isozymes I (CA I) and II (CA II) as protein model systems, having a broad spectrum of inhibitors that bear a primary sulfonamide group and exhibit various affinities.^40^ Carbonic anhydrases are a family of proteins catalyzing the hydration of carbon dioxide to bicarbonate and acid protons and thus performing functions related to the acid–base balance and carbon metabolism in the human body. Several CA isoforms are overexpressed in tumors and thus are investigated as cancer targets.^41−44^ Despite their vital role in many biological processes, there are very little data in the scientific literature on volume changes that occur when these proteins bind various ligands. The lack of volumetric data limits the application of these methods as standard techniques used in the development of new compounds as drug candidates.

In this manuscript, we exploit high-pressure NMR spectroscopy to determine the changes in the CA I and CA II volumes upon binding two ligands. We present an alternative approach to obtain the volumetric parameters from two-dimensional (2D) ^1^H–^15^N heteronuclear single quantum coherence (HSQC) spectra. The proposed analysis can significantly decrease the number of NMR spectra that are required to determine the protein–ligand binding volume.

## Materials and Methods

Expression of recombinant human CA I and CA II was performed in *Escherichia coli* strain BL21(DE). The overnight culture (10 mL) was grown in LB medium with 0.060 mM ZnCl_2_, then harvested by centrifugation (5 min, 4000*g*), and resuspended in M9 minimal nutrition medium (containing 42.2 mM (6 g/L) Na_2_HPO_4_, 22 mM (3 g/L) KH_2_PO_4_, 2 mM MgSO_4_, 0.10 mM CaCl_2_, and a mixture of trace metals) with 1 g/L ^15^N-labeled ammonium chloride, 4 g/L d-glucose, 0.060 mM ZnCl_2_, and 0.1 g/L ampicillin. The culture was grown at 37 °C, 220 rpm, for approximately 6 h until the optical density at 600 nm reached 0.6–0.8, and the protein expression was induced at this point with 0.20 mM IPTG and 0.4 mM ZnCl_2_ was added. The culture was harvested by centrifugation after 16 h of protein expression at 20 °C, 220 rpm.

CA I and CA II were purified using nickel-immobilized metal affinity chromatography (chelating Sepharose Fast Flow) and ion-exchange chromatography (SP-Sepharose column for CA I and CM-Sepharose for CA II) as previously described.^45,46^ The purified proteins were dialyzed against 10 mM Bis-Tris buffer at pH 6.2 (for CA I) and pH 6.4 (for CA II) and freeze-dried.

Protein samples of 0.52 mM or 0.43 mM CA I and 0.34 mM CA II were prepared in buffer (10 mM Bis-Tris, 50 mM NaCl, 10% D_2_O, 4% dimethyl sulfoxide (DMSO), at pH 6.2 for CA I and pH 6.4 for CA II) with different concentrations of compound **1** or **2**, which were prepared in pure DMSO.

High-pressure NMR spectroscopy was used to record 2D ^1^H–^15^N HSQC spectra of CA I and CA II at various pressures. The protein solution (0.33 mL) was added into a ceramic tube with an outer diameter of 5 mM and an inner diameter of 3 mM from Daedalus Innovations (Aston, PA). Hydrostatic pressure was applied to the sample directly within the magnet through an inox line filled with low-density paraffin oil (Sigma) using an Xtreme Syringe Pump from Daedalus Innovations. 2D ^1^H–^15^N HSQC spectra were recorded at 25 °C and eight different pressures ranging from 5 to 210 MPa on a Bruker AVANCE III 600 MHz equipped with a 5 mM Z-gradient TXI probe head. Water suppression was achieved using the WATERGATE method.^47^^1^H chemical shifts were directly referenced to the water resonance (4.7 ppm), while ^15^N chemical shifts were referenced indirectly to the ^15^N/^1^H absolute frequency ratios. Water resonance was used as the reference because the most commonly used referencing compound sodium trimethylsilylpropanesulfonate (DSS) might inhibit CA I and CA II. All NMR experiments were processed with TOPSPIN software (Bruker), and the spectra were analyzed with CcpNmr Analysis V2 software.^48^ A full set of spectra for one sample throughout the pressure range was recorded in approximately 20 h with CA I and 30 h with CA II protein samples.

The resonance assignment of human CA I was determined from previous studies^49^ and taken from Biological Magnetic Resonance Data Bank (entry number: 4022, doi:10.13018/BMR4022). The assignment of CA II was kindly shared by Ronald A. Venter from Duke University NMR Center.^50^

## Results and Discussion

**Figure 2:**
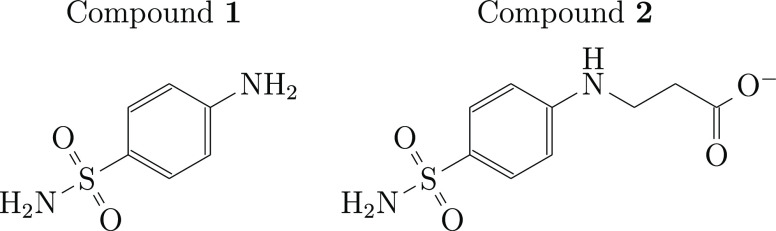
Compounds that were used to measure the protein–ligand binding volume.

**Figure 3:**
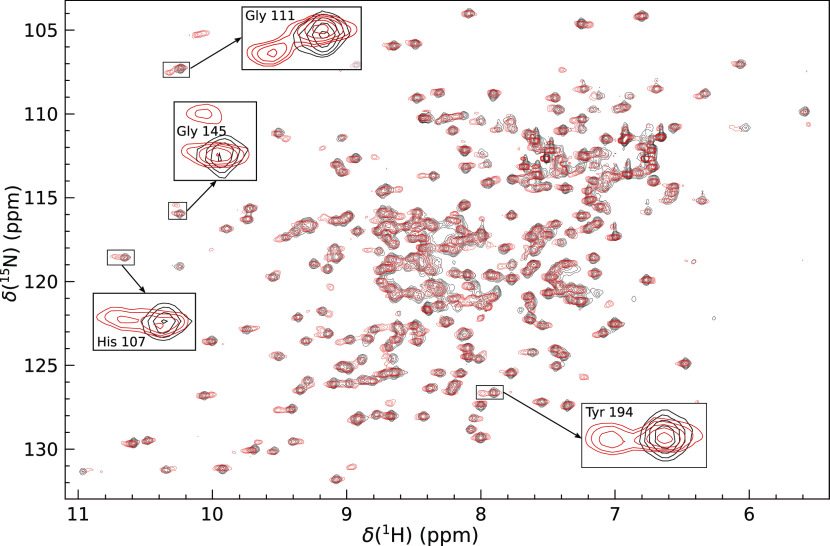
^1^H–^15^N HSQC spectrum overlay of CA I (0.52 mM) without a ligand (black) and with 0.70 mM compound **1** (red) at 5 MPa. Magnified peaks of His 107, Gly 111, Gly 145, and Tyr 194 amino acid residues highlight that in the presence of compound **1**, both ligand-bound and ligand-free states of the protein were observed in the spectrum of CA I. The resonance assignment was taken from doi:10.13018/BMR4022.

In this study, we used two compounds that have a primary sulfonamide group in their structures (Figure 2) and exhibit sub-millimolar binding affinities for CA I and CA II.^40,51^ Two-dimensional ^1^H–^15^N HSQC NMR spectra showed that both isoforms of carbonic anhydrase are in the slow exchange (in NMR time-scale) between two protein states—the ligand-bound and ligand-free—for compounds **1** and **2**. This exchange rate allowed the monitoring of peaks that correspond to ligand-free and ligand-bound amino acid residues of a protein in the same spectrum. The intensity ratio of ligand-free and ligand-bound peaks is proportional to the fraction of protein molar concentrations in each state.^39^ In the calculations, we used the volume of amide cross-peaks in ^1^H–^15^N HSQC spectra. Many residues showed a second peak corresponding to the ligand-bound state upon addition of either compound **1** or **2**. Figure 3 shows the cross-peaks of CA I affected by compound **1** in the overlaid ^1^H–^15^N HSQC spectrum at a pressure of 5 MPa.

**Figure 4:**
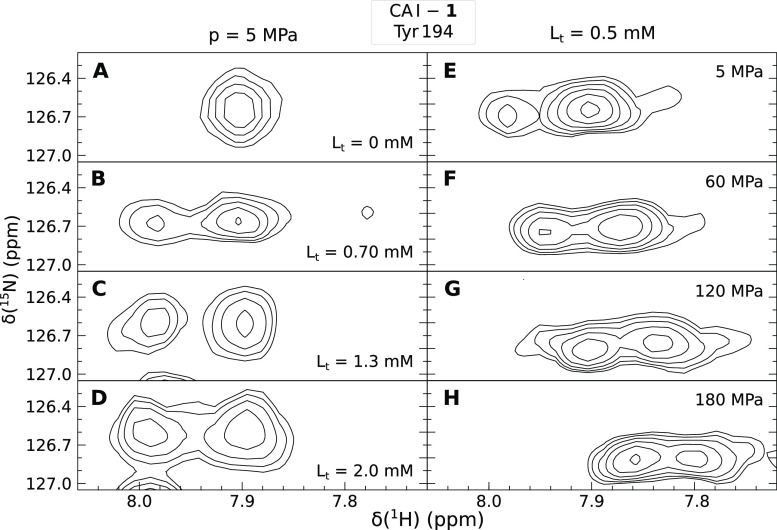
Ligand- and pressure-induced shifts of the Tyr 194 residue peak position in the ^1^H–^15^N HSQC spectra. The concentrations of Compound **1** ranged from 0 to 2.0 mM (left panels) and pressure values ranged from 5 to 180 MPa (right panels).

Increasing ligand concentrations enhance the intensity of the peak corresponding to the ligand-bound state of CA I. The left panels in Figure 4A–D show the increase in the intensity of the bound-state Tyr 194 peak of CA I at a fixed pressure. In this case, the positions of both peaks in the spectrum remained steady, and the changes occurred only in their intensities. Pressure can also be used to rearrange the intensity ratio of ligand-bound and ligand-free peaks. The right panels in Figure 4E–H show the behavior of the Tyr 194 peak at different pressures and a fixed concentration of the ligand. As the pressure increases, the intensity of the bound-state peak increases and both peaks propagate along δ(^1^H) and δ(^15^N) axes in the ^1^H–^15^N HSQC spectra. At elevated pressures, the protein–ligand system tries to occupy a lower volume (Le Chatelier’s principle). Thus, if increasing pressure enhances the peak intensity of the ligand-bound protein state, the partial volume of the protein–ligand complex is lower than that of the sum of individual partial volumes of the protein and the ligand.

**Figure 5:**
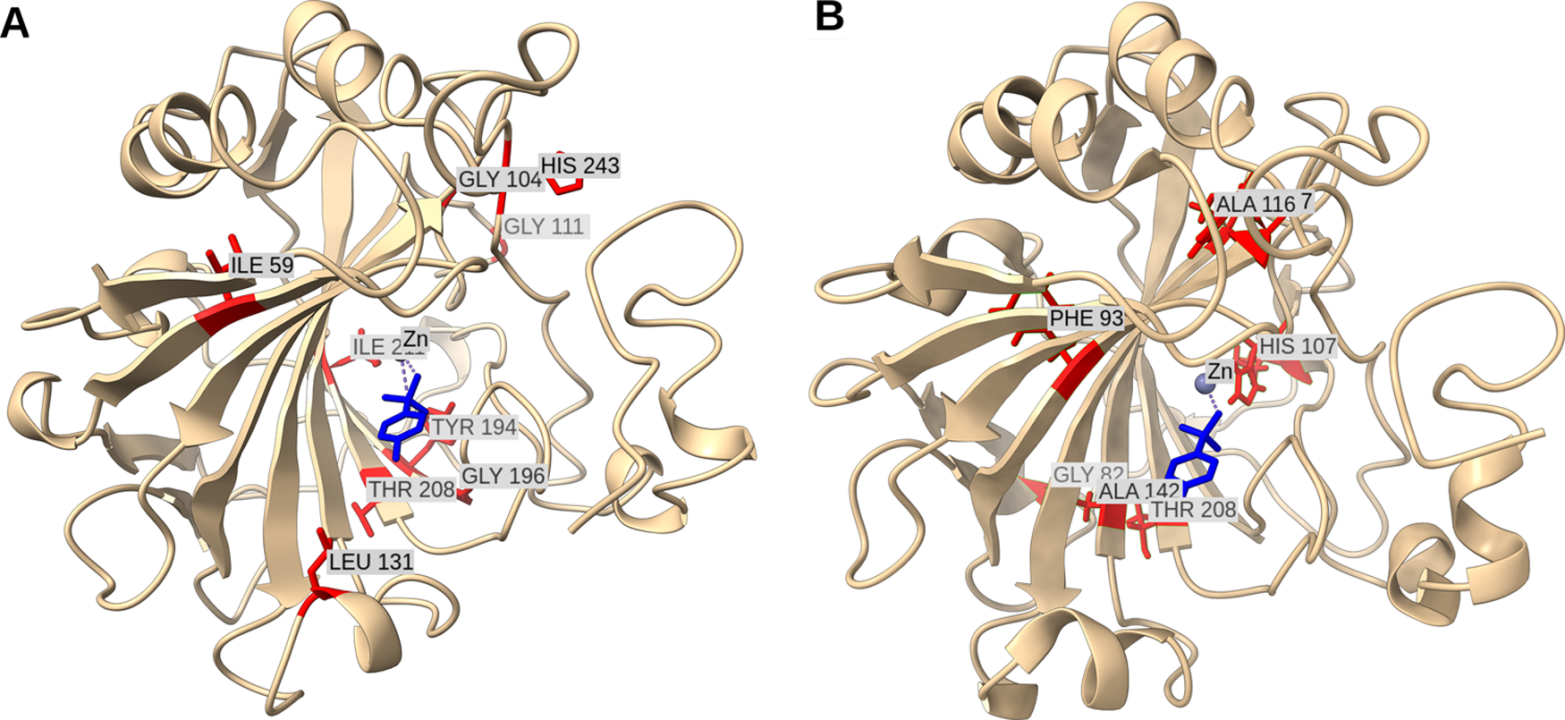
Crystal structures of the carbonic anhydrase complex with compound **1**: (A) CA I (PDB ID: 1CZM) and (B) CA II (PDB ID: 6RL9). Molecules of compound **1** are shown as blue sticks. Residues that were mostly affected by binding of **1** are colored red and labeled.

The fraction of the bound protein, θ_*i*_, was calculated from the volumes of amide cross-peaks of the ligand-bound (*I*_PL_(*i*)) and ligand-free (*I*_P_(*i*)) states of the *i*-th amino acid residue using the following equation
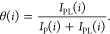
1Fluctuations of the peak intensity in the spectrum can lead to inaccurate values of the protein–ligand binding volume if a single amino acid is analyzed. We used an arbitrary ensemble of amino acid residues, which showed the peaks of ligand-bound and ligand-free protein states upon ligand binding. In further calculations, we used the ensemble-averaged fraction of the ligand-bound protein, θ
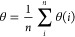
2where *n* is the number of amino acid residues in the ensemble, which was individually arranged for a particular pair of protein and ligand. Many residues in the ^1^H–^15^N HSQC spectra showed both ligand-bound and ligand-free forms of the protein, but some of the signals overlapped at high pressures and were hardly distinguishable, and thus, such residues were not used for further analysis. For example, we have chosen an ensemble of nine amino acid residues to monitor the CA I interaction with compound **1**: Ile 59, Gly 104, Gly 111, Leu 131, Tyr 194, Gly 196, Thr 208, Ile 211, and His 243. Figure 5 shows the positions of analyzed residues in the crystal structures of CA I (panel A) and CA II (panel B) complexes with compound **1**. Most ligand-binding-affected amino acid residues were near the active sites of both enzymes CA I and CA II. However, most probably due to remote rearrangements of the three-dimensional (3D) structure of the protein upon ligand binding, some more distant residues also showed a ligand-bound protein peak in the ^1^H–^15^N HSQC spectrum.

The change in volume in textbooks of Thermodynamics is defined as the partial derivative of the Gibbs energy with respect to pressure; thus, the change in volume upon protein–ligand binding at a constant temperature, *T*, is
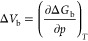
3where *p* denotes the pressure, and Δ*G*_b_ is the change in the standard Gibbs energy of binding (the standard state is defined as 1 mol/L concentrations of the participating substances at 1 bar pressure and the activity coefficients are equal to 1).^32^ This equation shows that to obtain Δ*V*_b_, we first need to determine Δ*G*_b_ at various pressures. In experiments, usually the dissociation equilibrium constant, *K*_d_, is determined, while the change in the Gibbs energy is calculated using the equation Δ*G*_b_ = *RT*  ln *K*_d_.

**Figure 6:**
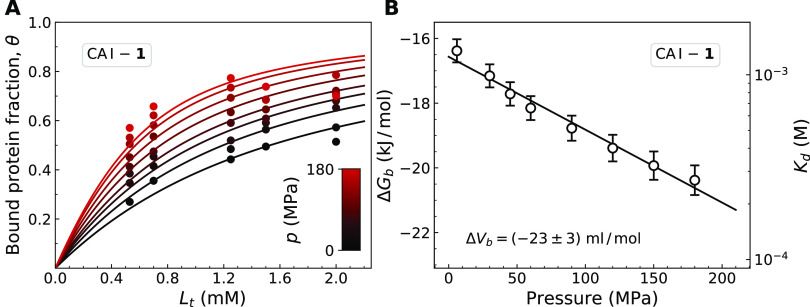
(A) Fractions of the ligand-bound CA I protein determined from ^1^H–^15^N HSQC spectra at different pressures. The inset shows a pressure scale with black color corresponding to low pressure and red color corresponding to high pressure. The concentrations of Compound **1** were 0.53, 0.70, 1.3, 1.5, and 2.0 mM. Solid lines show fits to experimental data using eq 4 that yielded *K*_d_ values at different pressures. (B) Calculated Δ*G*_b_ = *RT* ln(*K*_d_) plotted as a function of pressure. The error bars denote standard errors obtained from the nonlinear fitting of experimental data in panel A.

In this study, we aimed to show that Δ*V*_b_ can be obtained from ^1^H–^15^N HSQC spectra at various pressures using a single concentration of protein and ligand. To validate this method, we first determined Δ*V*_b_ values by titrating a protein solution with a ligand at different pressures. The first step of this approach requires to determine *K*_d_ from the fraction of ligand-bound protein with the increasing ligand concentration
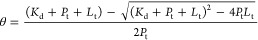
4where *P*_t_ and *L*_t_ denote total (initial) concentrations of the added protein and ligand, respectively. We used this equation in nonlinear regression analysis to determine protein–ligand dissociation constants (and subsequently Δ*G*_b_) at various pressures. Figure 6A shows experimental data and analyzed results of the CA I interaction with compound **1** at various pressures up to 180 MPa. The resulting Δ*G*_b_ as a function of pressure (Figure 6B) allows calculating the parameter of interest Δ*V*_b_. We assumed that the second pressure derivative of the Gibbs energy at a constant temperature is zero, implying that in the range of investigated pressures, Δ*V*_b_ is a pressure-independent parameter. This assumption allowed us to calculate Δ*V*_b_ from the slope of the linear fit to pressure dependence of Δ*G*_b_ (Figure 6B). The calculated Δ*V*_b_ of the CA I interaction with compound **1** was (−23 ± 3) mL/mol.

**Figure 7:**
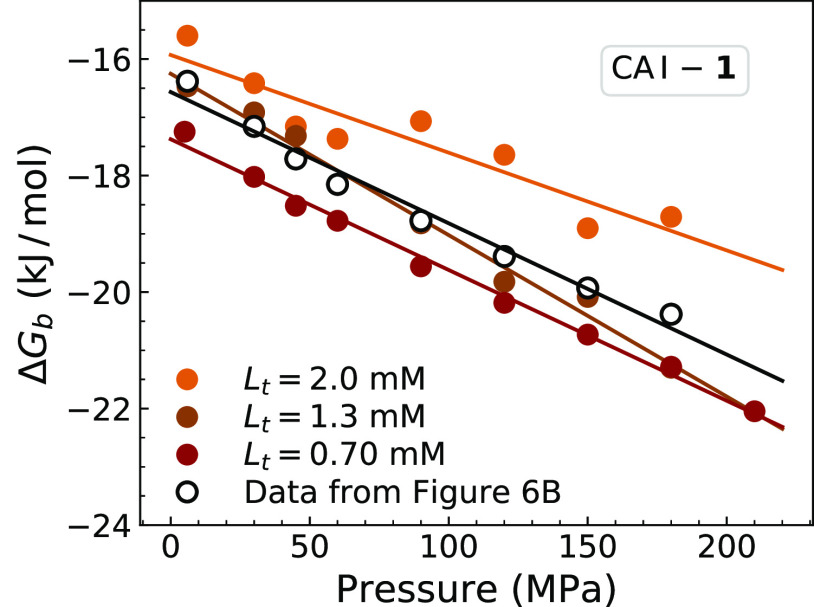
Comparison of Δ*V*_b_ calculations. Colored circles represent Δ*G*_b_ values calculated from ^1^H–^15^N HSQC spectra of CA I that were recorded at 0.70, 1.3, and 2.0 mM concentrations of the added ligand. Data shown as open circles were replotted from Figure 6B to compare different approaches to obtain Δ*G*_b_s.

The described method allows the determination of *K*_d_ as a function of pressure, which is necessary to obtain Δ*V*_b_. However, this method is time-consuming and requires relatively large amounts of a protein and a ligand. The slow chemical exchange between the ligand-free state of carbonic anhydrases (CA I, CA II) and the ligand-bound state with compounds **1** and **2** allowed an alternative approach to determine *K*_d_ using a single concentration of protein and ligand at various pressures. In this case, *K*_d_ can be determined using the rearranged definition of the dissociation constant , where [L], [P], and [PL] denote the molar concentrations of the free ligand, the unbound protein, and the ligand-bound protein, respectively. We used the mass balance condition *P*_t_ = [PL] + [P] = [PL](1 + [P]/[PL]) and expressed as . We obtained the final equation by substituting the [PL] term into the rearranged *K*_d_ equation
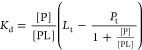
5The ratio  is the intensity ratio of the peaks corresponding to the ligand-free and ligand-bound states of a particular amino acid residue in the ^1^H–^15^N HSQC spectrum. Equation 5 is used to determine *K*_d_s as a function of pressure, using a single concentration of the added protein and ligand. Figure 7 shows the resulting plots of Δ*G*_b_ versus pressure, which were obtained from several single-concentration experiments. To compare results from different methods, in Figure 7, we also plotted Δ*G*_b_ dependence on pressure as shown in Figure 6. This comparison shows that different approaches to determine *K*_d_ yielded similar Δ*G*_b_ dependencies on pressure.

**Figure 8:**
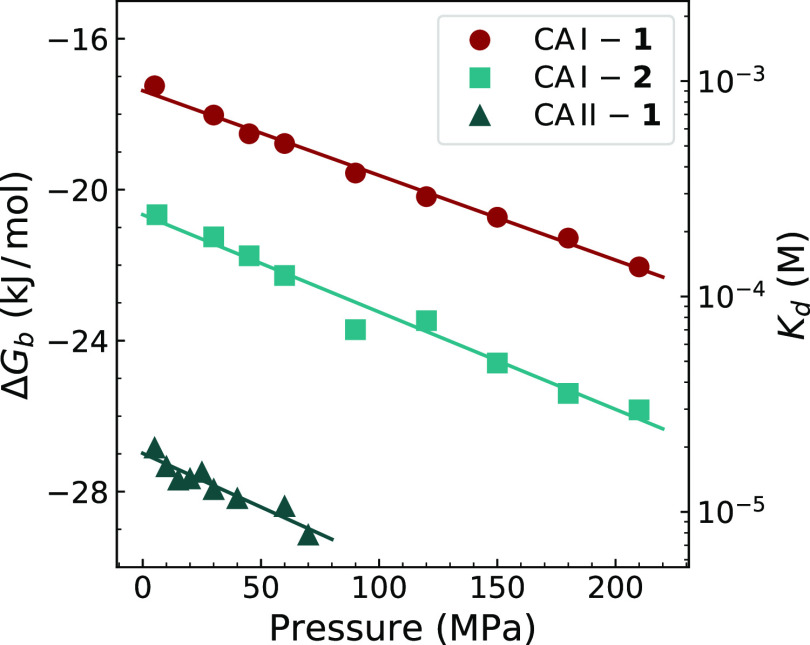
Change in the Gibbs energy of the protein–ligand binding versus applied pressure at a fixed concentration of the added ligand. Δ*G*_b_ values were obtained from the intensity ratio of peaks in the ^1^H–^15^N HSQC spectrum corresponding to ligand-free and ligand-bound states of the protein and averaged over an ensemble of binding-affected residues. The scale on the right shows *K*_d_ values that correspond to the Δ*G*_b_ scale at *T* = 25 °C.

Gibbs energies of CA I and CA II binding to compounds **1** and **2** were calculated for a set of ligand binding-affected protein residues in the ^1^H–^15^N HSQC spectra at different pressures. Figure 8 shows averaged Δ*G*_b_ values as a function of pressure. To determine Δ*G*_b_ values, we used information from the protein ^1^H–^15^N HSQC spectra recorded at 0.7 mM (CA I-1), 0.6 mM (CA I-2), and 0.4 mM (CA II-**1**) concentrations of the added ligand. A decrease in Δ*G*_b_ with increasing pressure means negative protein–ligand binding volumes. Linear data fits yielded the Δ*V*_b_ equal to (−22 ± 4) mL/mol for CA I-1, (−26 ± 4) mL/mol for CA I-2, and (−28 ± 4) mL/mol for CA II-**1**. The uncertainty given next to the Δ*V*_b_ values shows the standard deviation, which was evaluated by comparing Δ*V*_b_ values at different concentrations of the added ligand.

## Discussion

We have described a method to determine the protein–ligand binding volume using a series of ^1^H–^15^N HSQC spectra recorded at several different pressures and using a single concentration of the added ligand. Besides the benefits of determining Δ*V*_b_ with less NMR spectrometry time, this approach could also raise questions about reliability. To examine this, we have determined Δ*V*_b_ values at several fixed concentrations of the added ligand. The most accurate Δ*V*_b_ values were obtained when the molar concentration of the added ligand was slightly less than the dissociation constant of the protein–ligand interaction. Such ligand concentrations allow one to expect that both unbound and bound protein populations coexist at a range of pressure values. If the initial ligand concentrations exceed the *K*_d_, the peak intensity of the ligand-bound protein state starts to dominate over the ligand-free state and its peak intensity cannot be determined accurately from the ^1^H–^15^N HSQC spectra. Moreover, the intensity of peaks corresponding to the ligand-free protein state decreases further because increasing pressure promotes the protein–ligand binding and diminishes the population of the unbound protein state.

**Figure 9:**
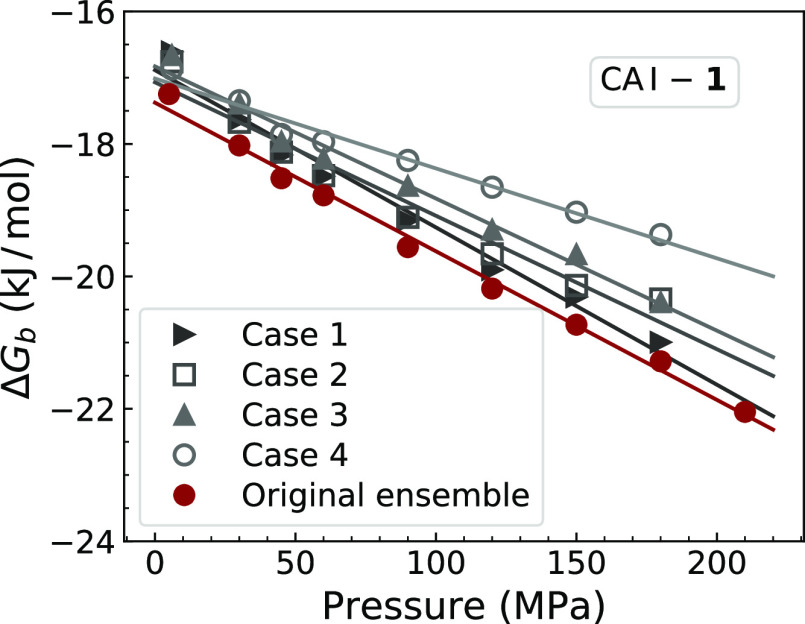
Comparison of calculated Δ*V*_b_ values of the CA I-**1** interaction, if analysis was performed on the reduced ensembles of ligand binding-affected residues. Cases 1–4 are described in the text.

Another potential problem regarding the accuracy of Δ*V*_b_ is related to the ensemble size and particular selection of amino acid residues for the analysis. Although many peaks in the ^1^H–^15^N HSQC spectrum showed both ligand-bound and ligand-free forms of protein, not all of them were suitable for analysis. The doubling of peaks in the ^1^H–^15^N HSQC spectrum due to the slow exchange of ligand-free and ligand-bound states complicated the data analysis as some peaks overlapped with each other. This problem forced us to select only clearly visible peaks. We selected groups consisting of 9–13 residues to calculate dissociation constants of the investigated protein–ligand pairs at various pressures. We also questioned, how would Δ*V*_b_ values change if the number of residues in the ensemble was reduced or selected improperly. To test this, we simulated four case scenarios with reduced ensembles of binding-affected amino acid residues. In CA I-**1** analysis, we excluded (1) several more distant residues with respect to the center of the ligand-binding site, (2) all amino acids except nonpolar ones, (3) all Gly residues, and (4) all Gly residues in addition to the second case scenario. Figure 9 shows that these trials resulted in Δ*V*_b_ values that were similar to those obtained by averaging over the initial ensemble of residues. Case 4 showed the biggest deviation since only three out of nine residues were left after the applied criteria. These tests showed that Δ*V*_b_ values were less sensitive to the selection of ensemble than to the too high or too low concentration of the added ligand.

A notable advantage of NMR spectroscopy to determine volume-related properties of proteins is its capability to provide simultaneous information about environmental changes of the binding-affected amino acids. This knowledge could reveal the regions of a protein that contribute mostly to the overall change in volume upon binding a ligand. The ^1^H–^15^N HSQC spectrum at different pressures also gives insight into compression properties at various sites of the protein and allows one to determine whether the low-lying excited states are populated at high pressures.^17^ Compressibility is a thermodynamic parameter that reveals the nonlinear response of the Gibbs energy to the applied pressure. It is difficult to obtain this parameter experimentally and very little data are available in the literature about protein compressibility changes upon binding a ligand.^33,52−54^ It is desirable to determine the change in compressibility by analyzing the corresponding volume changes. In this study, the most Δ*G*_b_ dependencies on pressure demonstrated a linear decrease in the range of investigated pressures. This could mean that CA I and CA II compressibility changes were very small upon binding compounds **1** and **2**. Another possible explanation could be averaging over an ensemble of residues, which might obscure the nonlinear response of a particular residue. Although nonlinear regression analysis could be used to evaluate compressibilities in these protein–ligand systems, we decided not to account for them. Every additional parameter in the nonlinear regression analysis increases the uncertainty and makes fitted parameters less reliable. This problem becomes especially relevant if the parameter of interest describes weakly expressed nonlinear effects, as was the case in our study. Therefore, we assumed that Δ*V*_b_ was a pressure-independent parameter in our calculations.

**Figure 10:**
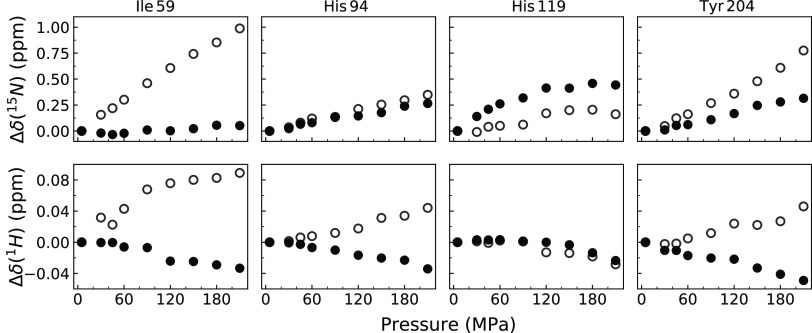
Response of CA I (0.52 mM) residues to the applied pressure in the absence (open circles) and presence (solid circles) of compound **1** (0.70 mM).

Although compressibility was not accounted for in the volume calculation, we analyzed residues showing three different responses to the applied pressure. Ile 59 and Tyr 204 residues of CA I were the most pronounced examples showing distinct linear responses to pressure in ligand-bound and ligand-free protein states. Linear chemical shift variations are due to structural fluctuations within the basic folded state of the protein. In the case of amide chemical shifts, they are essentially related to H-bond compressibility.^55^ Ligand-free state peaks of both residues experienced relatively high (compared to all residues) chemical shift change, Δδ, in the ^15^N dimension at 210 MPa. The Δδ(^15^N) was approximately 10 times lower for the CA I-**1**-bound state of Ile 59 and up to 3 times lower for the CA I-**1**-bound state of the Tyr 204 residue compared to that of the ligand-free state of CA I (Figure 10). This result might indicate that compound **1** reduces the volume fluctuations of Ile 59 and Tyr 204 residues in CA I. Similar behavior was observed for CA II amino acid residues Ile 33 and Phe 93. This effect could be either due to the direct ligand interaction with these amino acid residues or the change in protein conformation upon binding compound **1**. The reduced volume fluctuations (which are related to protein flexibility) have been reported in several studies of the protein–ligand interaction.^35,56−58^ As an example, the study of dihydrofolate reductase showed that compressibility is related to enzyme reactivity—the protein–substrate complex was the most compressed (tight structure), but after the product was formed the compression reduced (more flexible or soft state).^53^

The Val 62, Arg 89, and Leu 147 residues in CA I were examples of the second type of response to high pressures. These residues showed the highest linear Δδ, but their responses to pressure were similar for both the ligand-bound and ligand-free CA I states. This suggests high volume fluctuations in these regions that are independent of compound **1** binding to CA I. The chemical shift change of Val 62 is nonlinear up to 210 MPa pressure, suggesting conformational change and possibly populated low-lying excited states of this amino acid residue at a high pressure. Nonlinear chemical shift variations are due to structural fluctuations involving low-lying excited states that differ from the basic folded states.^55^

His 94 and His 119 showed the third type of response to high pressures. These residues together with His 96 coordinate the Zn ion in the active sites of CA I and CA II. The amide nitrogen chemical shift change of His 94 in CA I-**2** was slightly higher at high pressures (not shown in Figure 10) and His 119 in the CA I-**1**-bound state, compared with that in the ligand-free protein state. This suggests that the change in hydrogen bonds (dependent on the ^1^H chemical shift change) or dihedral angles of the peptide bond (dependent on ^15^N chemical shift change) was larger for the ligand-bound state. This was not a clear case for His 94 in the CA I-**1** complex state shown in Figure 10. However, the chemical shift changes of these residues were close to the average (Δδ(^15^N) = 0.4 ppm at 210 MPa) and could not be used to account for CA I and CA II compressibility changes upon binding a ligand. Interestingly, the proton chemical shifts of His 94 and Tyr 204 move to different directions of the ligand-bound and ligand-free states of CA I with increasing pressure. This behavior could indicate that the hydrogen bond changes the length in opposite directions of the ligand-bound and ligand-free states (shorter bond in the case of negative chemical shift change and longer bond in the case of positive chemical shift change) with increasing pressure.^59,60^

The presented method to determine Δ*V*_b_ from one concentration pair of protein and ligand at several pressures could not be extended to the fast-exchange regime of the protein interaction with a ligand. In this regime, the ligand-free and ligand-bound states of the protein appear in one averaged peak due to fast-exchange rate compared to the chemical shift difference between these two states. Thus, it is not possible to determine the ratio of ligand-free and ligand-bound states of the protein from a single peak and one has to measure chemical shift changes upon ligand titration to the protein solution. If we could determine the fraction of the ligand-bound protein from one peak, which is a result of the fast-exchange between two states (it is possible if the dissociation constant is known), the peak position with increasing pressure would depend not only on the binding properties but also on the compressibility effect. These two effects would make the analysis difficult to determine Δ*V*_b_. The only way to obtain this parameter would be a titration experiment at different pressures, if protein and ligand are in a fast-exchange regime and the compressibility remains unchanged in the ligand-free and ligand-bound protein states.

Despite the discussed limitations, the proposed analysis enables us to determine Δ*V*_b_ in many protein–ligand systems obeying the described conditions. The obtained Δ*V*_b_ values of the CA I and CA II interaction with the ligands were within the range of −22 to −28 mL/mol. The binding volume of −32 mL/mol of the CA I interaction with the other compound bearing a primary sulfonamide group in its structure—acetazolamide—was determined previously by a fluorescent pressure shift assay.^61^ Acetazolamide has a higher affinity toward CA I^40^ and more negative Δ*V*_b_ than structurally similar compounds **1** and **2**. These results were compatible with previous findings showing correlations between protein–ligand binding volumes and affinities.^29^ To the best of our knowledge, no other values of Δ*V*_b_ for the family of carbonic anhydrases were reported in the literature. Other globular protein and ligand systems showed rather similar values of Δ*V*_b._^25^

## Conclusions

The analysis of 2D ^1^H–^15^N HSQC spectra of the protein–ligand interaction with increasing pressure enabled us to determine the change in the protein volume upon binding a ligand. If ligand-free and ligand-bound protein states were in a slow-exchange regime in the NMR time-scale, the protein–ligand binding volume could be obtained from a single pair of protein and ligand at several increasing pressures.
